# Multi-Objective Optimization of the Dry Towpreg Filament Winding Process for Carbon/Epoxy Type IV Hydrogen Storage Vessels

**DOI:** 10.3390/polym18050639

**Published:** 2026-03-05

**Authors:** Ruiqi Li, Kaidong Zheng, Xiaoyu Yan, Haonan Liu, Yu Zhang, Guangming Huo, Haixiao Hu, Dongfeng Cao, Hao Li, Hongda Chen, Shuxin Li

**Affiliations:** 1 State Key Laboratory of Advanced Technology for Materials Synthesis and Processing, Wuhan University of Technology, Wuhan 430070, China; ricky_lee@whut.edu.cn (R.L.);; 2 National Energy Key Laboratory for New Hydrogen-Ammonia Energy Technologies, Foshan Xianhu Laboratory, Foshan 528200, China; whut_zkd@163.com (K.Z.);; 3 Hubei Key Laboratory of Theory and Application of Advanced Materials Mechanics, School of Physics and Mechanics, Wuhan University of Technology, Wuhan 430070, China

**Keywords:** carbon fiber composites, dry towpreg filament winding, type IV hydrogen storage vessel, multi-objective optimization, hydrogen storage efficiency

## Abstract

Hydrogen storage vessels are critical components in hydrogen energy systems, and improving their manufacturing efficiency and structural performance is essential for next-generation Type IV vessel designs. Compared with conventional wet filament winding, towpreg dry filament winding offers higher efficiency, reduced environmental impact, and better adaptability to complex structures. In this study, key process parameters, including winding tension, heating temperature, and winding speed were systematically optimized using the tensile strength and interlaminar shear strength of NOL ring specimens as evaluation metrics. A response surface methodology (RSM) regression model was established to correlate process variables with mechanical properties, followed by multi-objective optimization using the non-dominated sorting genetic algorithm II (NSGA-II) and final parameter selection through the Technique for Order Preference by Similarity to Ideal Solution (TOPSIS) method. The results indicate that shear strength is primarily affected by heating temperature, whereas tensile strength is mainly governed by winding tension. The optimal parameter combination (79 N, 360 °C, and 11 m/min) yielded tensile and shear strengths of 2462.2 MPa and 64.4 MPa, respectively, with prediction errors below 0.5%. A 9 L Type IV hydrogen storage vessel manufactured under these conditions showed approximately 15.4% lower mass and about 17% higher gravimetric hydrogen storage efficiency than a comparable wet wound vessel.

## 1. Introduction

Filament winding is a widely used composite manufacturing technique due to its simple process, low cost, and capability to fabricate components with complex geometries. It has been extensively applied in aerospace, automotive, and new energy industries [1,2]. In the field of hydrogen energy, hydrogen storage vessels serve as the core storage units of hydrogen-powered systems, and their hydrogen storage density directly determines the driving range of the system. Compared with conventional wet filament winding, the dry towpreg filament winding not only retains the advantages of traditional filament winding but also enables a higher and more stable fiber volume fraction, thereby improving the overall performance of hydrogen storage vessels [3]. For filament-wound composite structures, mechanical performance depends not only on the intrinsic properties of fibers and resins but also strongly on manufacturing process parameters. Therefore, optimizing the filament winding process parameters is essential for enhancing the comprehensive performance of high-pressure hydrogen storage vessels.

Extensive studies have been conducted on the mechanical behavior of filament-wound carbon fiber composites [4,5,6,7,8,9]. Furthermore, increasing attention has been paid to the influence of process parameters on filament winding performance. Deng et al. [10] investigated the prepreg tape winding process using NOL ring tensile strength as the optimization objective and analyzed the effects of heating temperature, winding tension, and roller pressure through response surface methodology. Khan et al. [11,12] developed simulation tools for the prepreg tape placement process to study the effects of placement speed, temperature, and pressure on product quality. Supian [13] experimentally investigated the influence of winding angle on the impact resistance of filament-wound composites. Zu et al. [14] simulated the application of winding tension using an equivalent cooling method and analyzed optimal tension schemes through finite element modeling. Mansingh [15] systematically studied the effects of winding tension on porosity and tensile properties by combining numerical simulations with experimental validation. Blachut et al. [16] examined the influence of winding tension on the burst pressure of metal-lined composite pressure vessels and found that increased winding tension induces compressive stress in the liner, thereby enhancing burst strength. Sara [17] employed response surface methodology (RSM) to investigate the effects of winding speed, winding angle, and winding tension on the tensile and compressive properties of wet wound composite pipes. Mohammadi et al. [18] compared wet filament winding with towpreg winding and demonstrated that the towpreg process significantly improves interlaminar shear strength, fiber-direction tensile strength, and transverse tensile strength. Schmidt et al. [19] optimized wet winding parameters by minimizing porosity through a combination of NOL ring testing and microscopic analysis. Orman et al. [20] optimized the filament winding parameters of thermoplastic composites using a Box–Behnken design, with compressive strength as the optimization target.

Based on the impregnation state of fibers prior to winding, filament winding processes can be classified into towpreg dry winding, wet winding, and semi-dry winding [21]. Current research has mainly focused on mature technologies such as wet filament winding and prepreg tape placement. However, studies on dry towpreg filament winding, which has attracted increasing attention in recent years, remain relatively limited. Semi-dry prepreg offers advantages such as high production efficiency, simplified process flow, and precise control of resin content. In addition, the reduced relative slippage during winding makes this process particularly suitable for manufacturing components with complex geometries, demonstrating strong potential in hydrogen storage pressure vessels [22]. Nevertheless, due to significant differences in resin impregnation, tow delivery, and spreading mechanisms of material, existing optimization studies cannot be directly applied to dry towpreg filament winding. Moreover, filament-wound products often require the simultaneous optimization of multiple performance, making optimization of key process parameters remain challenge.

In this study, a multi-objective optimization of the dry towpreg filament winding process for Type IV hydrogen storage vessels was conducted. Winding tension, heating temperature, and winding speed were selected as the key process parameters, while the mechanical properties including tensile strength and interlaminar shear strength of NOL ring specimens were used as the primary performance indicators. First, regression models describing the relationships between process parameters and mechanical properties were established and evaluated based on RSM. Subsequently, multi-objective optimization was performed using the Non-dominated Sorting Genetic Algorithm II (NSGA-II) to obtain the Pareto-optimal solution set. The optimal process parameter combination was then determined using the Technique for Order Preference by Similarity to Ideal Solution (TOPSIS) and experimentally validated. On this basis, the effect mechanisms of process conditions on mechanical performance were analyzed through composites morphology observation. Finally, Type IV hydrogen storage vessels were manufactured using the optimized towpreg dry winding process and compared with conventional wet filament wound vessels with the same winding pattern through burst pressure tests, providing a comprehensive evaluation of the advantages of dry towpreg filament winding.

## 2. Materials and Methods

This study systematically investigated the dry towpreg filament winding process using carbon fiber towpreg through mechanical testing of NOL ring specimens, combined with multi-objective optimization methods to determine the optimal process parameters. The optimized parameters were subsequently applied to the fabrication of Type IV hydrogen storage vessels, followed by burst pressure validation. The experimental procedure mainly comprised three stages: preparation and mechanical testing of composite specimens, multi-objective optimization of process parameters, and fabrication and burst testing of hydrogen storage vessels. The materials, equipment, and testing methods are described in detail below.

### 2.1. Materials and Equipment

The carbon fiber towpreg used in this study was supplied by Jiangsu Hengshen Co., Ltd. (Zhenjiang, China), with the designation SS91-30F. The carbon fiber mass fraction was 73wt%, and the matrix was an epoxy resin system specifically designed for dry towpreg filament winding applications. Filament winding was performed using a six-axis, five-linkage CNC filament winding machine (model SLW01.10.S-700-5/2-5000-CNC) manufactured by Hunan Jiangnan Sileng CNC Machinery Co., Ltd. (Xiangtan, China). The equipment is equipped with closed-loop tension control, real-time temperature monitoring, and adjustable winding speed, fully satisfying the requirements of dry towpreg filament winding. The curing process was conducted in a programmable rotary curing oven to ensure uniform temperature distribution throughout the specimens.

**Figure 1:**
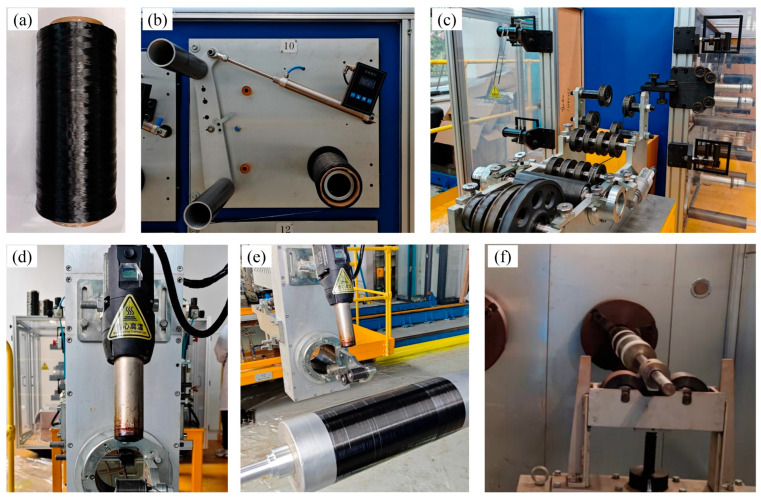
Dry towpreg filament winding process: (**a**) Prepreg material; (**b**) Tension control system; (**c**) Prepreg delivery system; (**d**) Hot air heating; (**e**) Cylindrical winding; (**f**) Curing.

For comparison, wet filament winding specimens were fabricated using the same type of carbon fiber (TZ700S-24K). The resin system for wet winding was an epoxy resin (EzCiclo RB240) supplied by Swancor Advanced Materials Co., Ltd. (Shanghai, China), with a mass ratio of 100:100 between resin (component A) and curing agent (component B). The dry towpreg filament winding process and experimental setup are illustrated in Figure 1, including the towpreg material, tension control system, towpreg delivery device, hot-air heating unit, cylindrical winding process, and curing stage.

### 2.2. Composite Specimen Preparation

Composite NOL ring specimens with thicknesses of 3.0 mm and 1.5 mm were fabricated using the dry towpreg filament winding process by controlling the winding tension, heating temperature, and winding speed. During winding, the carbon fiber towpreg was softened by the combined action of the tension control system and hot-air heating before being wound onto the mandrel surface. After winding, the vessels were cured in a rotary oven following the curing cycle of holding at 80 °C for 2 h and subsequently at 105 °C for 3 h.

**Figure 2:**
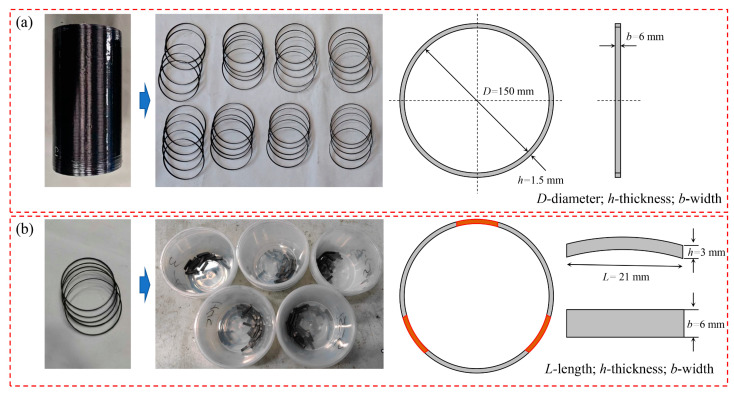
Schematic diagram of NOL ring specimens: (**a**) NOL ring tensile specimen; (**b**) NOL ring shear specimen.

For the preparation of NOL ring specimens, conventional approaches typically involve winding carbon fibers directly into grooves of a disk-shaped mandrel. However, this method often results in thickness fluctuations at the fiber start and end points, leading to increased scatter in mechanical test results [23]. To reduce the influence of thickness non-uniformity caused by fiber initiation and termination, an improved preparation method was adopted in this study. Cylindrical specimens with uniform thickness were first fabricated according to the required NOL ring thickness. Standard NOL ring specimens were then cut from the uniformly thick middle region of the vessels using numerical control machining. The specimen dimensions complied with the requirements specified in GB/T 1458–2023 [24] for filament-wound composite ring specimens subjected to tensile and shear testing. The geometries and dimensions of the tensile and shear NOL ring specimens are shown in Figure 2.

### 2.3. Mechanical Testing of Composite Specimens

**Figure 3:**
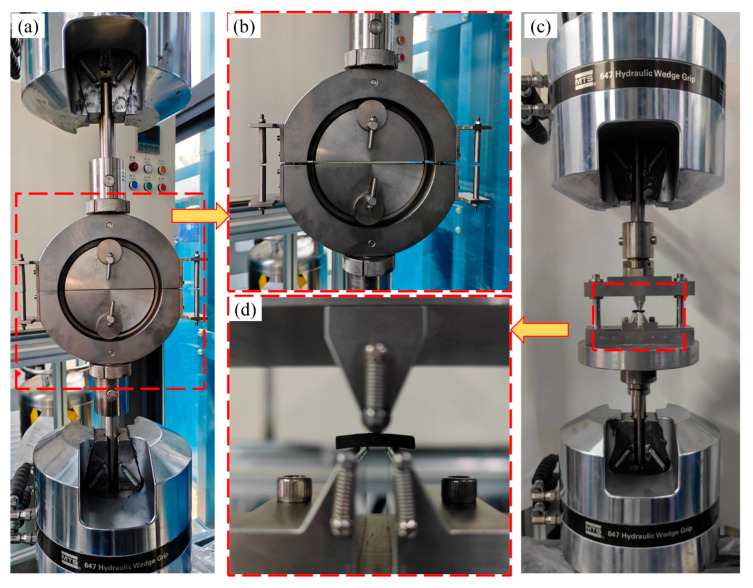
NOL ring tensile and shear tests: (**a**) Schematic diagram of NOL ring tensile test; (**b**) Close-up of NOL ring tensile test; (**c**) Schematic diagram of NOL ring shear test; (**d**) Close-up of NOL ring shear test.

The interlaminar shear strength and tensile strength of the NOL ring specimens were measured in accordance with GB/T 1458–2023. Tensile tests were conducted using a dedicated ring-shaped fixture to ensure uniform load application along the fiber direction, while shear tests were performed using a double-shear fixture to determine the interlaminar shear strength. Schematics of the testing configurations are presented in Figure 3.

For each set of process parameters, five valid specimens were prepared and tested. The average value of the measured results was used to reduce experimental scatter and improve data reliability.

### 2.4. Fabrication of Type IV Hydrogen Storage Vessels

To verify the engineering applicability of the optimized dry towpreg filament winding process, Type IV hydrogen storage vessels were fabricated using both the optimized dry winding parameters and a conventional wet winding process. The two fabrication routes employed identical winding patterns and tension schemes to ensure structural comparability.

For the dry towpreg filament winding vessels, the carbon fiber towpreg described above was wound onto a plastic liner using the optimized process parameters, followed by the same curing cycle described in Section 2.2. For the wet filament winding vessels, the same carbon fiber was impregnated through a resin bath maintained at 35 °C and subsequently wound onto the liner. The epoxy resin system used was EzCiclo RB240, and the curing cycle consisted of holding at 80 °C for 2 h followed by 105 °C for 5 h.

**Figure 4:**
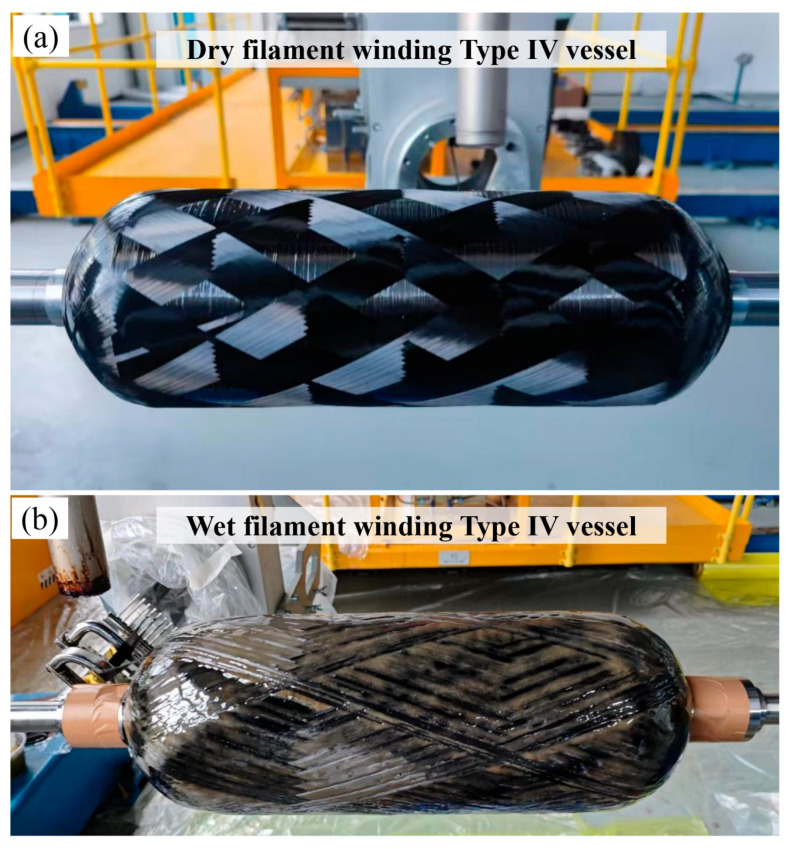
Fabrication of composite hydrogen storage vessels: (**a**) Dry filament-wound vessel; (**b**) Wet filament-wound vessel.

After winding and curing, all vessels were subjected to hydrostatic burst tests to determine the burst pressure and failure modes, which were used to evaluate the ultimate load-bearing capacity and structural reliability of the vessels. The fabrication procedures and representative specimens are shown in Figure 4.

## 3. Multi-Objective Optimization Method

### 3.1. Range of Process Parameters

The tensile strength and interlaminar shear strength of dry filament-wound composites are influenced by multiple process parameters, among which winding tension, heating temperature, and winding speed are the most critical. The mechanical performance of dry filament-wound composites is governed by factors such as fiber–resin interfacial bonding quality, fiber packing density, and resin curing uniformity, all of which are directly affected by the combined action of winding tension, heating temperature, and winding speed.

To ensure that the multi-objective optimization was conducted within a feasible and practical processing window, the ranges of the three key parameters were determined based on the material characteristics of the carbon fiber towpreg, equipment limitations, and preliminary experimental investigations.

Winding tension directly affects fiber compaction and fiber damage during the winding process. As shown in Figure 1b, the tension controller regulates the tension applied to the fiber bundle through the elastic arm of the creel, which is then transmitted to the mandrel surface through the tow delivery system. Excessively low winding tension results in insufficient fiber compaction on the mandrel, leading to interlaminar gaps, increased porosity, and degraded interlaminar properties. Conversely, excessively high tension may exceed the allowable stress of the carbon fibers in the towpreg, intensify fiber abrasion during tow delivery, and induce fiber damage or even filament breakage during winding. Based on experimental measurements, the winding tension range was set to 50–110 N.

Heating temperature plays a critical role in regulating the viscosity of the resin system and the activity of the curing agent in the towpreg. As illustrated in Figure 1d, the heating nozzle is positioned approximately 10 cm from the tow and the fiber guide, and hot air is used to heat both the tow and the nozzle, enabling the towpreg to soften prior to deposition on the mandrel. Insufficient heating temperature prevents adequate resin softening and viscosity reduction, resulting in poor fiber impregnation and weak interlaminar bonding. In contrast, excessively high temperatures may cause resin accumulation at the nozzle and premature local curing, leading to tow embrittlement, surface fuzzing, and even tow breakage, which severely compromises the mechanical properties of the final composite. Accordingly, the heating temperature range was determined to be 120–360 °C.

Winding speed is closely related to production efficiency and the duration of thermal–mechanical interaction during winding. Excessively low winding speeds prolong the exposure time of the resin to high temperatures, increasing the risk of premature curing and tow adhesion, while significantly reducing manufacturing efficiency. Excessively high winding speeds may exceed the stable operating limits of the equipment and control system, causing tension fluctuations, non-uniform fiber placement, and insufficient thermal exposure for adequate resin softening and impregnation. Therefore, the winding speed range was set to 5–17 m/min.

The ranges of the three process parameters are summarized in Table 1 and were used as factor levels in the response surface experimental design based on the Box–Behnken Design (BBD), ensuring both engineering feasibility and theoretical significance of the optimization study.

### 3.2. Multi-Objective Optimization of the Dry Towpreg Filament Winding Process

The key performance indicators of towpreg dry filament-wound composites are tensile strength and inter-laminar shear strength, which jointly determine the overall mechanical performance of the final products. These properties are primarily influenced by winding tension, heating temperature, and winding speed. In this study, tensile strength and shear strength were selected as the optimization objectives, and the three aforementioned process parameters were optimized simultaneously to determine the optimal processing window for dry towpreg filament winding.

**Figure 5:**
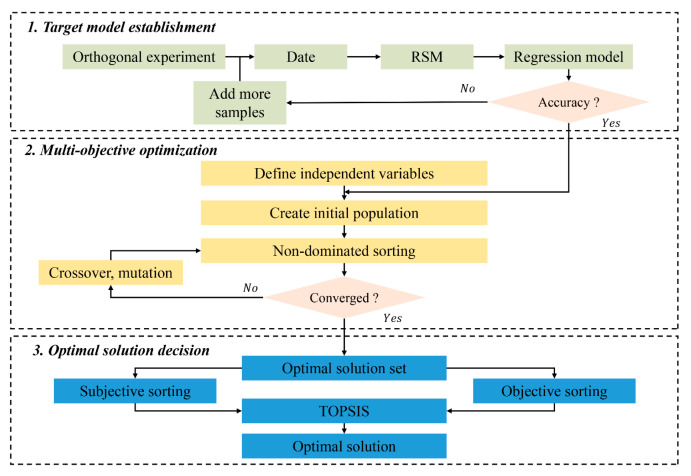
Framework of the multi-objective optimization method.

The overall framework of the multi-objective optimization strategy is illustrated in Figure 5. First, RSM was employed to construct regression models describing the relationships between winding tension, heating temperature, winding speed, and the tensile and shear strengths. Second, these regression models were used as objective functions in a multi-objective evolutionary optimization algorithm to generate a Pareto-optimal solution set. Finally, a multi-criteria decision-making method based on the TOPSIS was applied to select the optimal combination of process parameters from the Pareto front.

#### 3.2.1. Construction of RSM

RSM was employed to establish a data-driven optimization database. Regression models for the objective functions were developed using Design-Expert software (version 13). A Box–Behnken Design (BBD) was adopted to ensure accurate analysis and efficient utilization of experimental data. The second-order polynomial model was chosen because it is the standard form in RSM for capturing curvature and interaction effects among variables. Preliminary experiments indicated nonlinear relationships between process parameters and mechanical properties, justifying the use of a quadratic model. Moreover, the Box–Behnken design is specifically intended for fitting a second-order response surface, as it provides an efficient estimation of the quadratic terms without requiring a full three-level factorial design.

The three-factor, three-level experimental design and corresponding results for the dry towpreg filament winding process are presented in Table 2, where winding tension, heating temperature, and winding speed were treated as independent variables, and the shear strength and tensile strength of the NOL ring specimens were considered as response variables. Based on the experimental results, regression analyses were performed using Design-Expert to establish RSM function models with shear strength and tensile strength as the respective optimization objectives.

#### 3.2.2. Multi-Objective Optimization Based on the NSGA-II Algorithm

Based on the established regression models, a multi-objective optimization was conducted with shear strength and tensile strength as the optimization objectives. In conventional optimization approaches, the two objective functions, shear strength $R_{1}$(A,B,C) and tensile strength $R_{2}$(A,B,C), cannot be maximized simultaneously. The multi-objective optimization algorithm adopted in this study coordinates the trade-off between the objective functions and generates a set of optimal solutions in which each solution represents a compromise between competing objectives. This set of solutions is referred to as the Pareto-optimal solution set.

**Figure 6:**
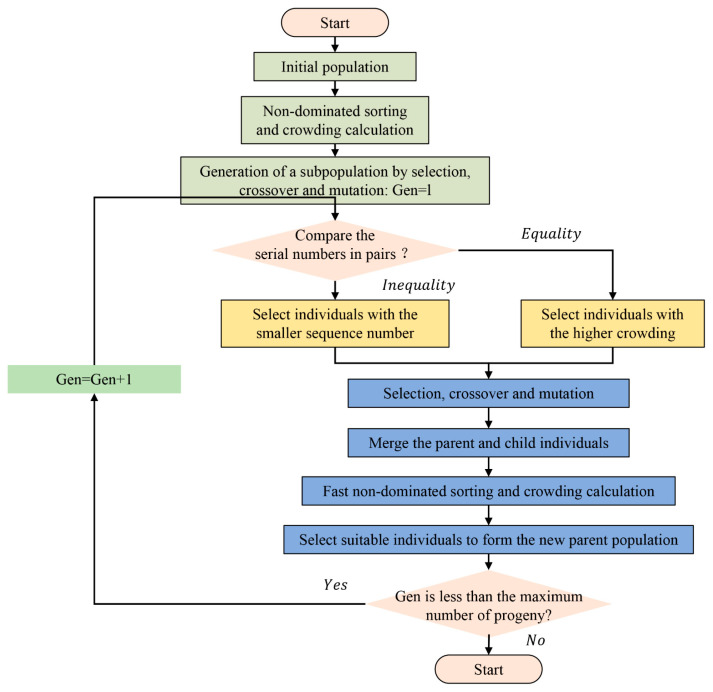
Optimization process of the NSGA-II genetic algorithm.

NSGA-II is a widely used multi-objective optimization algorithm characterized by fast convergence, high computational efficiency, and strong robustness, making it suitable for solving complex multi-objective optimization problems. The optimization procedure of the NSGA-II algorithm is illustrated in Figure 6.

In this study, the dry towpreg filament winding process was optimized by maximizing both shear strength $R_{1}$ and tensile strength $R_{2}$. Considering practical manufacturing constraints, the process parameters—winding tension A, heating temperature B, and winding speed C—were treated as bounded variables rather than continuous variables. Based on the above considerations, a multi-objective optimization model for the NOL ring preparation process was formulated using the NSGA-II algorithm, subject to the following constraints:

(1) $$\begin{array}{c} maxF\left(A,B,C\right)=\left[maxR_{1},maxR_{2}\right]\\ s.t.\left\{\begin{array}{c} 50\leq A\leq 110,A\in N\\ 120\leq B\leq 360,B\in 10\;N\\ 5\leq C\leq 17,C\in N \end{array}\right. \end{array}$$

The NSGA-II algorithm was implemented using custom MATLAB (version 2020b) code. The number of decision variables was set to three, and the number of objective functions was set to two. The population size was 50, the maximum number of generations was 200, the crossover probability was 0.8, and the mutation probability was 0.05. The optimization procedure involved population initialization, non-dominated sorting, crowding distance calculation, selection, crossover, and mutation. After iterative evolution, the Pareto-optimal solution set was obtained.

#### 3.2.3. Multi-Objective Decision-Making Based on TOPSIS with Game Theory Weighting

The NSGA-II genetic algorithm was used for multi-objective optimization, resulting in a set of Pareto-optimal solutions. This solution set includes multiple solutions that balance the different optimization objectives. The selection of the optimal solution involves subjective judgment based on engineering feasibility and the relative importance of each optimization objective. This section evaluates the importance of NOL ring shear strength and tensile strength and calculates the subjective weights of the two optimization objectives. The objective weights for the optimization goals were then calculated using the COWA operator. Based on game theory, the combined weights of the optimization objectives were computed, and a weighting matrix was constructed. Finally, the TOPSIS method was applied to calculate the closeness index $R_{m}$ for each solution in the Pareto-optimal set, and the optimal solution was selected. The specific methods and related formula derivations are outlined below.

The subjective weights of NOL ring shear strength and tensile strength were calculated using the expert scoring method, with the formula:

(2) $$\omega_{1n}=\frac{\sum_{i=1}^{g}A_{in}}{\sum_{i=1}^{g}\sum_{n=1}^{2}A_{in}}$$

where $\omega_{1n}$ is the subjective weight of the n-th evaluation indicator; $A_{in}$ is the score given by the i-th expert for the n-th indicator; g is the number of experts involved in scoring.

The optimization objectives, i.e., NOL ring shear strength and tensile strength, were taken as evaluation indicators, resulting in the following indicator matrix S:

(3) $$S=\left[\begin{array}{cc} s_{11} & s_{12}\\ \vdots & \vdots \\ s_{m1} & s_{m2}\\ \vdots & \vdots \\ s_{t1} & s_{t2} \end{array}\right]$$

where m is the index of a single solution in the Pareto set, and t is the total number of solutions in the set.

The elements of the indicator matrix $s_{mn}$ were normalized to obtain the standardized elements $s_{mn}^{*}$ and the standardized indicator matrix $S^{*}$:

(4) $$S^{*}=\left[\begin{array}{cc} s_{11}^{*} & s_{12}^{*}\\ \vdots & \vdots \\ s_{m1}^{*} & s_{m2}^{*}\\ \vdots & \vdots \\ s_{t1}^{*} & s_{t2}^{*} \end{array}\right]$$

(5) $$s_{mn}^{*}=\frac{s_{mn}}{s_{max}}$$

where $s_{max}$ is the maximum value of the n-th indicator.

The COWA method is an objective weighting approach that reduces the impact of extreme values in the Pareto-optimal solution set by reordering the evaluation values. The objective weights $\omega_{2n}$ for shear strength and tensile strength were calculated using the COWA operator:

(6) $$\omega_{2n}=\frac{\omega_{2n}^{*}}{\sum_{n=1}^{2}\omega_{2n}^{*}}$$

The formula for calculating $\omega_{2n}^{*}$ is:

(7) $$\omega_{2n}^{*}=\sum_{m=1}^{t}v_{m}\cdot s_{mn}^{*}$$

where

(8) $$v_{m}=\frac{C_{t-1}^{m-1}}{\sum_{u=0}^{t-1}C_{t-1}^{u}}=\frac{C_{t-1}^{m-1}}{2^{t-1}}$$

Here, $C_{t-1}^{m-1}$ represents the number of ways to choose m−1 elements from t−1 elements.

The game theory-based combination weighting method is a technique for coordinating different weight vector sets. This method compares and coordinates the subjective weight vector set $W_{1}=\{\omega_{11},\omega_{12}\}$ and the objective weight vector set $W_{2}=\{\omega_{21},\omega_{22}\}$. The linear combination coefficients $a=\{a_{1},a_{2}\}$ are defined, and the linear combination of the subjective and objective weight vectors is expressed as:

(9) $$\varphi =\sum_{j=1}^{2}a_{j}\cdot W_{j}$$

The coefficients $a_{j}$ are optimized to minimize the deviation between φ and $W_{j}$. The linear system of equations for the first-order optimization condition, based on matrix differentiation properties, is:

(10) $$\left[\begin{array}{cc} W_{1}\cdot W_{1}^{T} & W_{1}\cdot W_{2}^{T}\\ {W_{2}\cdot W}_{1}^{T} & W_{2}\cdot W_{2}^{T} \end{array}\right]\left[\begin{array}{c} a_{1}\\ a_{2} \end{array}\right]=\left[\begin{array}{c} W_{1}\cdot W_{1}^{T}\\ W_{2}\cdot W_{1}^{T} \end{array}\right]$$

From this, $a_{j}$ is calculated and normalized to obtain $a_{j}^{*}$:

(11) $$a_{j}^{*}=\frac{a_{j}}{\sum_{j=1}^{2}a_{j}}$$

The combined weights for the optimization objectives, based on the game theory method, are calculated as:

(12) $$\varphi_{n}=\sum_{j=1}^{2}a_{j}^{*}\cdot \omega_{jn}$$

The standardized indicator matrix $S^{*}$ is weighted to obtain the weighted matrix K:

(13) $$K=\left[\begin{array}{cc} k_{11} & k_{12}\\ \vdots & \vdots \\ k_{m1} & k_{m2}\\ \vdots & \vdots \\ k_{t1} & k_{t2} \end{array}\right]$$

where:

(14) $$k_{mn}=s_{mn}^{*}\cdot \varphi_{n}$$

The positive and negative ideal solutions for each optimization objective are determined in the weighted matrix:

(15) $$Y_{n}^{+}=min(k_{1n},k_{2n},\cdots ,k_{tn})$$

(16) $$Y_{n}^{-}=max(k_{1n},k_{2n},\cdots ,k_{tn})$$

The Euclidean distance $Z_{m}^{+}$ and $Z_{m}^{-}$ of each element in the weighted matrix K to the positive ideal solutions $Y_{n}^{+}$ and negative ideal solutions $Y_{n}^{-}$ are calculated as:

(17) $$Z_{m}^{+}=\sqrt{\sum_{n=1}^{2}{\left(k_{mn}-Y_{n}^{+}\right)}^{2}}$$

(18) $$Z_{m}^{-}=\sqrt{\sum_{n=1}^{2}{\left(k_{mn}-Y_{n}^{-}\right)}^{2}}$$

The closeness index $R_{m}$ for each solution in the Pareto-optimal set to the ideal solution is calculated as:

(19) $$R_{m}=\frac{Z_{m}^{-}}{Z_{m}^{+}+Z_{m}^{-}}$$

The value of $R_{m}$ lies between 0 and 1, where a higher value indicates that the solution is closer to the optimal solution. The solutions are ranked in descending order of $R_{m}$, and the corresponding sample with the highest $R_{m}$ is selected as the multi-objective decision result.

## 4. Results and Discussions

### 4.1. Multi-Objective Optimization of the Towpreg Filament Winding Process

#### 4.1.1. Response Surface Regression Models for Process Parameters

Based on the three-factor, three-level Box–Behnken experimental design, winding tension A, heating temperature B, and winding speed C were selected as independent variables, while the shear strength and tensile strength of NOL ring specimens were used as response variables. The experimental results are summarized in Table 2. Regression analysis was performed using Design-Expert software to establish response surface models correlating the process parameters with the mechanical properties of the NOL rings.

The resulting second-order polynomial regression models for shear strength $R_{1}$ and tensile strength $R_{2}$ can be expressed as Equation (20):

(20) $$R_{1}=\left\{\begin{array}{c} \begin{array}{rrr} 49.06115 & \cdot & \\ +0.119222 & \cdot & A\\ +0.011402 & \cdot & B\\ +0.369861 & \cdot & C\\ +0.000015 & \cdot & AB\\ -0.000014 & \cdot & AC\\ +0.000062 & \cdot & BC\\ -0.000474 & \cdot & A^{2}\\ +0.000014 & \cdot & B^{2}\\ -0.016563 & \cdot & C^{2} \end{array} \end{array}\right.,R_{2}=\left\{\begin{array}{c} \begin{array}{rrr} 1865.603 & \cdot & \\ +16.05053 & \cdot & A\\ +0.123828 & \cdot & B\\ -0.340972 & \cdot & C\\ -0.000505 & \cdot & AB\\ +0.002771 & \cdot & AC\\ -0.002155 & \cdot & BC\\ -0.112672 & \cdot & A^{2}\\ +4.727020 & \cdot & B^{2}\\ +0.008223 & \cdot & C^{2} \end{array} \end{array}\right.$$

#### 4.1.2. Analysis of the Regression Models

(1) Shear Strength Regression Model $R_{1}$ Analysis

**Figure 7:**
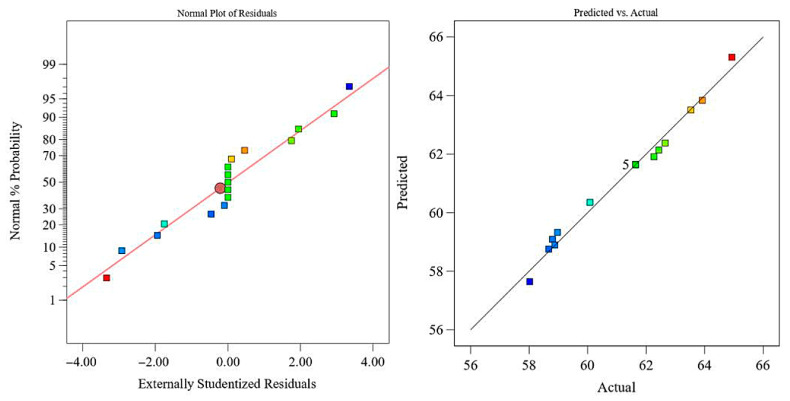
Reliability analysis of the shear strength regression model $R_{1}$.

In the analysis of variance (ANOVA), the F-value is used to evaluate the significance of differences among groups, while the *p*-value indicates the statistical significance of the regression model. A larger F-value and a smaller *p*-value correspond to a more significant model and a better fitting accuracy. As shown in Table 3, the shear strength regression model $R_{1}$ exhibits an F-value of 58.51 with a *p*-value lower than 0.0001, indicating that the model is statistically significant. The F-values of winding tension *A*, heating temperature *B*, and winding speed *C* decrease in the order F_B_, F_A_, F_C_. This demonstrates that heating temperature *B* has the most pronounced influence on the interlaminar shear strength of NOL rings, followed by winding tension *A*, while winding speed *C* has the least effect. The *p*-value associated with the linear term of winding speed exceeds 0.05, indicating a statistically insignificant linear effect. However, the quadratic term of winding speed shows a *p*-value of 0.0104, suggesting a nonlinear relationship between winding speed and shear strength. The coefficient of determination $R^{2}$ of the shear strength model is 0.9869, and the adjusted ${R^{2}}_{Adj}$ is 0.9700, indicating a high level of model reliability and predictive capability. Therefore, the regression model can be effectively used to analyze and predict the shear strength of towpreg dry filament-wound composites. It is worth noting that although some interaction terms (e.g., AB, AC, and BC) in Table 3 exhibit relatively high *p*-values, all terms were retained in the final regression model to maintain model hierarchy and ensure consistency with the tensile strength model for comparative purposes. This practice aligns with standard RSM, where model hierarchy is preserved even when certain terms appear statistically insignificant. The adequacy of the model was further confirmed by the high adjusted R^2^ value of 0.9700 and the residual analysis presented in Figure 7, both of which indicate no evidence of overfitting and support the reliability of the model for prediction within the experimental range.

Residual analysis of the shear strength regression model $R_{1}$ is shown in Figure 7. Except for a few outliers, the residuals are approximately normally distributed along a straight line, confirming the normality assumption of the regression model $R_{1}$. A comparison between the predicted and experimental values further demonstrates close agreement, indicating that the model exhibits high accuracy and robustness.

**Figure 8:**
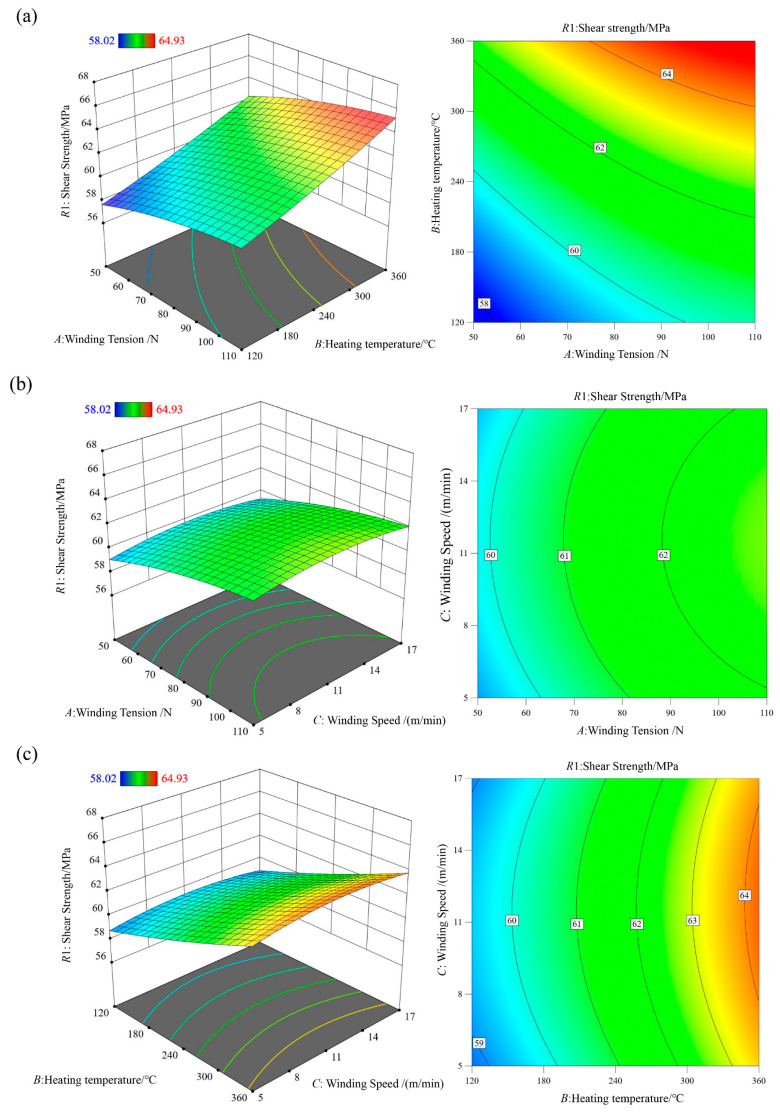
Interaction effects of different process conditions on the shear strength function $R_{1}$: (**a**) Winding tension *A*—Heating temperature *B*; (**b**) Winding tension *A*—Winding speed *C*; (**c**) Heating temperature *B*—Winding speed *C*.

The three-dimensional response surfaces and contour plots illustrating the interaction effects of process parameters on shear strength are shown in Figure 8. Among the parameters, heating temperature *B* exhibits the most significant influence, followed by winding tension *A*. Shear strength increases with increasing heating temperature *B* and winding tension *A*, whereas it initially increases slightly and then decreases with increasing winding speed *C*.

(2) Tensile Strength Regression Model $R_{2}$ Analysis

**Figure 9:**
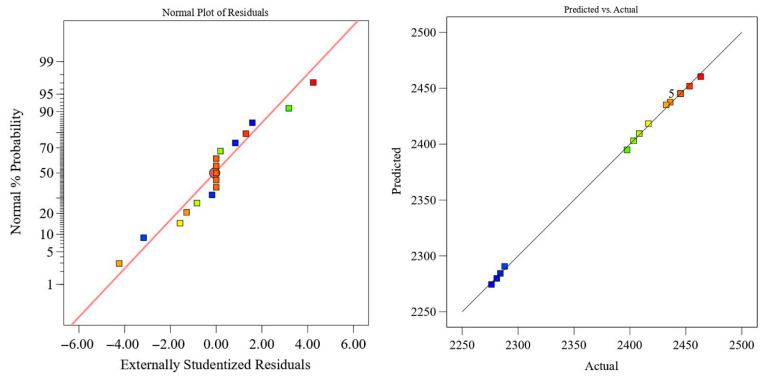
Reliability analysis of the tensile strength regression model $R_{2}$.

The ANOVA results for the tensile strength regression model are presented in Table 4. The model exhibits an F-value of 1489.5 with a *p*-value lower than 0.0001, confirming its high statistical significance. The F-values of winding tension *A*, heating temperature *B*, and winding speed *C* decrease in the order F_A_, F_B_, F_C_, indicating that winding tension is the dominant factor affecting tensile strength. The *p*-value associated with winding speed is 0.0166, suggesting a statistically significant linear relationship between winding speed and tensile strength. The coefficient of determination $R^{2}$ and adjusted ${R^{2}}_{Adj}$ of the tensile strength model are 0.9994 and 0.9987, respectively, demonstrating excellent fitting accuracy and predictive reliability. Following the same modeling approach as for shear strength, all terms were retained in the tensile strength regression model to maintain model hierarchy and ensure comparability between the two response surfaces. The high adjusted R^2^ value of 0.9987 and the residual analysis (Figure 9) confirm the model’s predictive capability and rule out overfitting concerns, even though some interaction terms in Table 4 exhibit relatively high *p*-values.

Residual analysis results for the tensile strength regression model are shown in Figure 9. The residuals are approximately normally distributed, and the predicted values are in close agreement with the experimental results, confirming the validity of the regression model.

**Figure 10:**
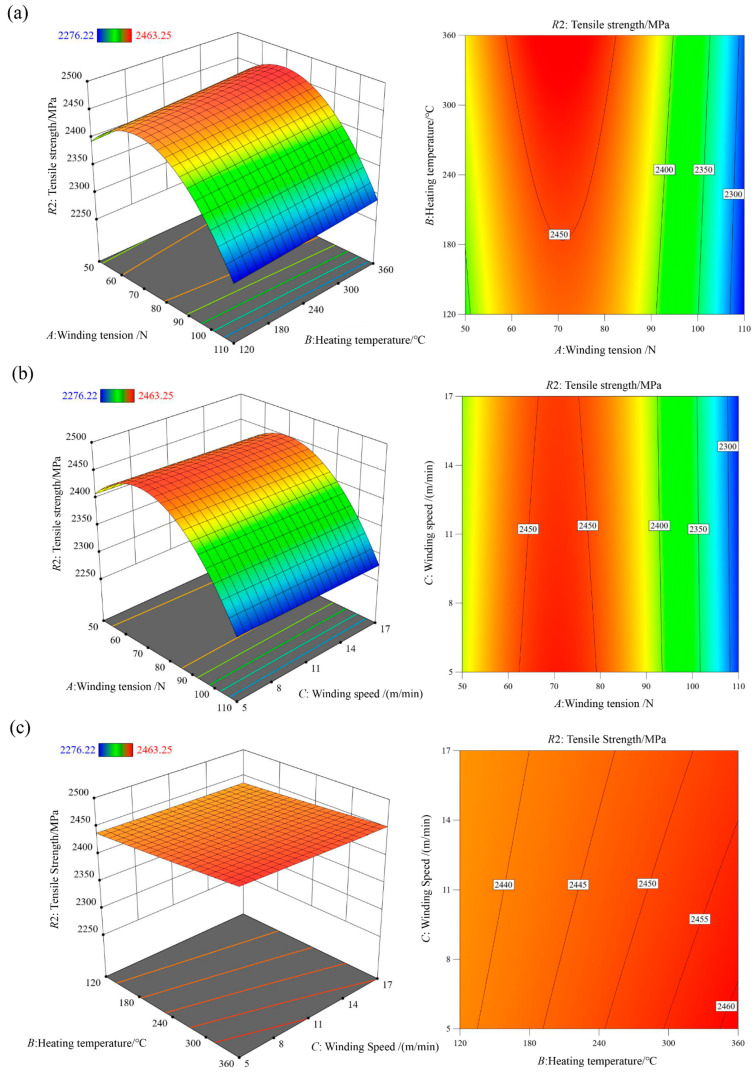
Interaction effects of different process conditions on the tensile strength regression model $R_{2}$: (**a**) Winding tension *A*—Heating temperature *B*; (**b**) Winding tension *A*—Winding speed *C*; (**c**) Heating temperature *B*—Winding speed *C*.

The interaction effects of process parameters on tensile strength are illustrated through three-dimensional response surfaces and contour plots in Figure 10. Winding tension *A* has the most pronounced effect on tensile strength, followed by heating temperature *B*, while winding speed *C* has a relatively minor influence. Tensile strength initially increases and then decreases with increasing winding tension *A*, increases slightly with heating temperature *B*, and decreases marginally with increasing winding speed *C*.

#### 4.1.3. Multi-Objective Optimization Results and Experimental Validation

**Figure 11:**
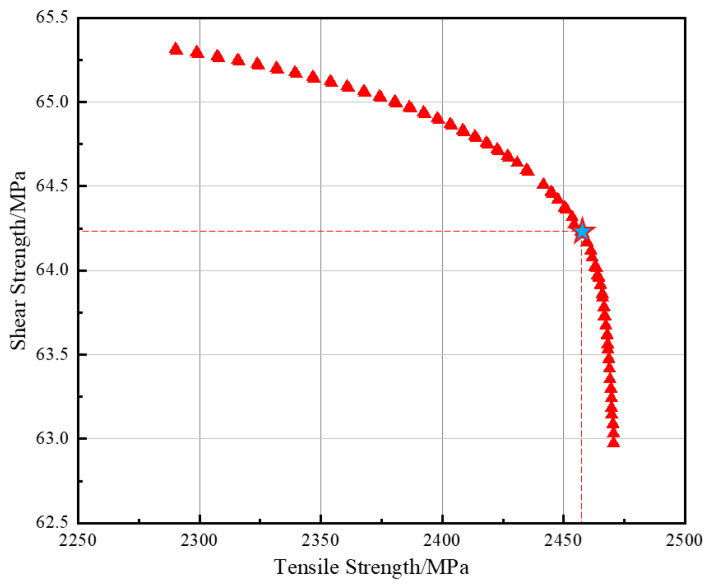
Pareto-optimal solution set and position of the solution ranked 1st on the Pareto front.

The NSGA-II algorithm was employed to perform multi-objective optimization with tensile strength and shear strength as the objective functions. The optimization parameters were set as follows: population size of 50, maximum number of generations of 200, crossover probability of 0.8, and mutation probability of 0.05. Through iterative evolution involving population initialization, non-dominated sorting, crowding distance calculation, selection, crossover, and mutation, a Pareto-optimal solution set consisting of 50 non-dominated solutions was obtained, as shown in Figure 11.

The subjective weights of the two evaluation indicators, NOL ring shear strength and tensile strength, were calculated using an expert scoring method. Ten experts in the relevant field assigned scores to the indicators, as shown in Table 5. The scoring scale corresponds to the following importance levels: 1 for “Not Important,” 2 for “Slightly Important,” 3 for “Moderately Important,” 4 for “Important,” and 5 for “Very Important.” The subjective weights for the NOL ring shear strength and tensile strength were then calculated using Equation (2), yielding values of $\omega_{11}=0.405$ and $\omega_{12}=0.595$, respectively. The subjective weights of NOL ring shear strength and tensile strength were calculated using the expert scoring method. A panel of 10 experts was invited to participate in the scoring process. The panel consisted of researchers and engineers specializing in composite materials, filament winding manufacturing, and hydrogen storage vessel design, each with 5–15 years of relevant experience in academia or industry.

The objective weights for the two evaluation indicators were calculated using Equation (6), resulting in $\omega_{21}=0.5$ and $\omega_{22}=0.5$. Based on game theory and the combination weighting method, the linear combination coefficients for the comprehensive weights of the two indicators were determined using Equation (11), yielding $a_{1}^{*}=1$ and $a_{2}^{*}=0$. Subsequently, the comprehensive weights of the two evaluation indicators were calculated using Equation (12), with values of $\varphi_{1}=0.405$ and $\varphi_{2}=0.595$, respectively.

The TOPSIS method was then applied to calculate the closeness coefficient of each Pareto-optimal solution to the ideal solution. The top ten solutions ranked by closeness coefficient are listed in Table 6. The solution ranked first exhibits shear strength and tensile strength values of 64.21 MPa and 2457.85 MPa, respectively, and its location on the Pareto front is illustrated in Figure 11.

The process parameters corresponding to the top-ranked solution were selected for experimental validation. The distributions of shear strength and tensile strength obtained from the validation experiments are shown in Figure 11, and a comparison between the predicted and experimental values is presented in Table 7. The prediction error for each mechanical property was calculated as the relative difference between the predicted value and the average experimental value, expressed as a percentage:

(21) $$Error=\frac{|\mathrm{Predicted}-{\mathrm{Experimental}}_{\mathrm{avg}}|}{\mathrm{Predicted}}\times 100\%$$

The average prediction errors for shear strength and tensile strength are approximately 0.31% and 0.18%, respectively, indicating excellent agreement between the optimization results and experimental measurements. Based on the optimization results, the optimal dry towpreg filament winding process parameters were determined as a winding tension of 79 N, a heating temperature of 360 °C, and a winding speed of 11 m/min. Under these conditions, the NOL ring specimens exhibited a tensile strength of 2462.2 MPa and a shear strength of 64.4 MPa.

### 4.2. Damage Mechanism Analysis of Towpreg Filament-Wound Composites

**Figure 12:**
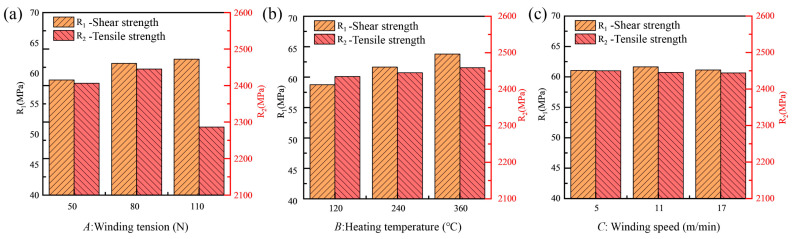
Effects of different process parameters on evaluation indicators: (**a**) Winding tension; (**b**) Heating temperature; (**c**) Winding speed.

Figure 12 illustrates the effects of winding tension, heating temperature, and winding speed on the shear strength and tensile strength of the composites. The results indicate that winding tension and heating temperature are the dominant parameters governing the towpreg state and final composite performance, whereas winding speed exerts a comparatively minor influence. To elucidate the underlying mechanisms, microscopic characterization was conducted to analyze the surface morphology and fracture behavior of composites produced under different winding tensions and heating temperatures. Within the selected winding speed range, variations in winding speed resulted in less than 5% fluctuation in mechanical properties. Therefore, its influence is not discussed in detail in the following sections.

#### 4.2.1. Effect of Winding Tension on Tensile Performance

**Figure 13:**
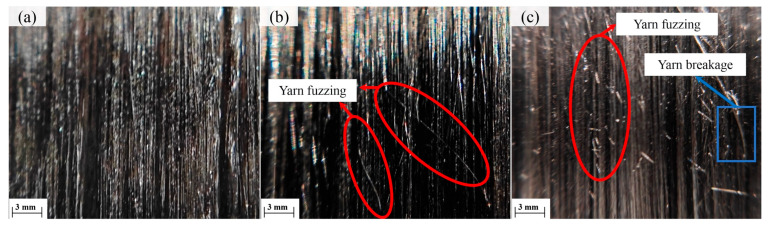
Surface morphology under different winding tensions during the winding process: (**a**) 50 N; (**b**) 80 N; (**c**) 110 N.

The multi-objective optimization results indicate that winding tension is the most significant factor influencing the tensile strength of NOL ring specimens. To investigate the underlying mechanism, the surface morphologies of composites fabricated under different winding tensions were examined. As shown in Figure 13, when the winding tension was 50 N, the composite surface exhibited uniform fiber distribution without noticeable fuzzing or filament breakage. When the tension increased to 80 N, slight fiber fuzzing began to appear. Further increasing the tension to 110 N resulted in pronounced fiber fuzzing accompanied by localized filament breakage. This behavior can be attributed to increased friction and abrasion during tow delivery and winding under excessive tension, which leads to fiber surface damage and filament fracture, ultimately degrading the tensile performance of the composite.

**Figure 14:**
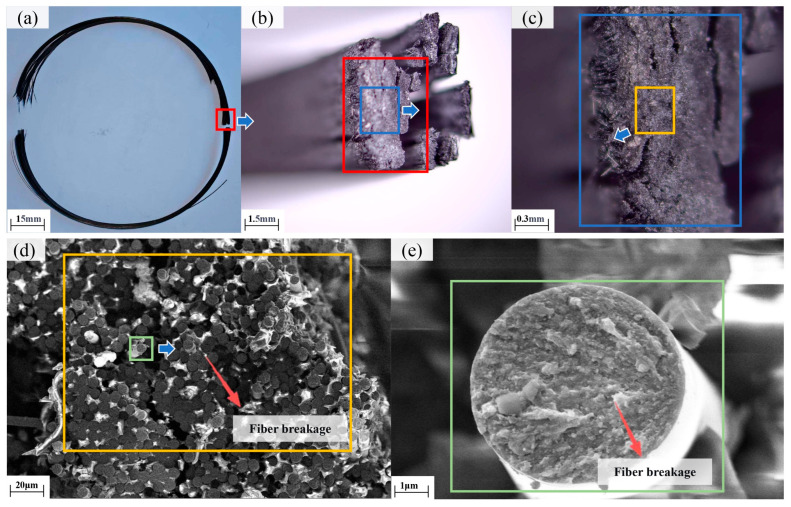
Tensile failure morphology of NOL ring: (**a**) Macroscopic; (**b**) Mesoscopic; (**c**) Mesoscopic magnification; (**d**) SEM; (**e**) High magnification SEM.

The fracture morphologies of tensile-tested NOL rings are shown in Figure 14. Tensile failure consistently occurred in the central cross-section of the specimens, with relatively flat fracture surfaces, consistent with failure modes observed in filament-wound pressure vessels subjected to hydrostatic burst tests [25,26]. Scanning electron microscopy (SEM) images of the fracture surfaces (Figure 14d,e) reveal that fiber fracture dominates the failure mechanism, with clean and aligned fracture surfaces. These observations indicate that tensile failure of NOL rings is primarily fiber-dominated. Consequently, excessive winding tension-induced fiber damage is identified as the primary cause of tensile strength degradation.

#### 4.2.2. Effect of Heating Temperature on Shear Performance

The optimization results demonstrate that heating temperature is the most influential parameter affecting the interlaminar shear strength of NOL ring specimens. Heating temperature directly governs resin flowability and impregnation behavior, thereby influencing fiber–resin interfacial bonding and defect distribution within the composite.

**Figure 15:**
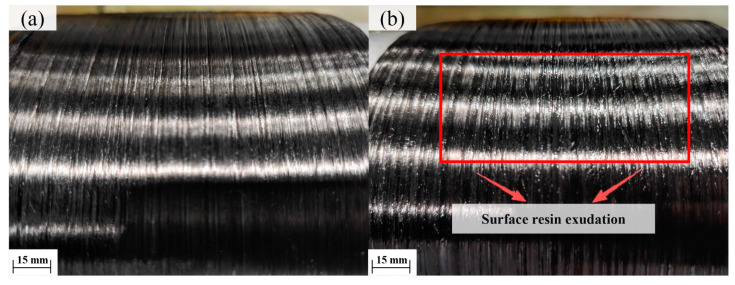
Surface resin exudation under different heating temperatures after winding: (**a**) 120 °C; (**b**) 360 °C.

Surface morphologies of composites fabricated at different heating temperatures are shown in Figure 15. At a heating temperature of 360 °C, noticeable resin exudation was observed on the composite surface, whereas specimens fabricated at 120 °C exhibited no obvious resin exudation and poorer surface smoothness. These results indicate that higher heating temperatures effectively soften the resin and enhance its flowability, promoting improved fiber wetting and stronger interlaminar bonding.

**Figure 16:**
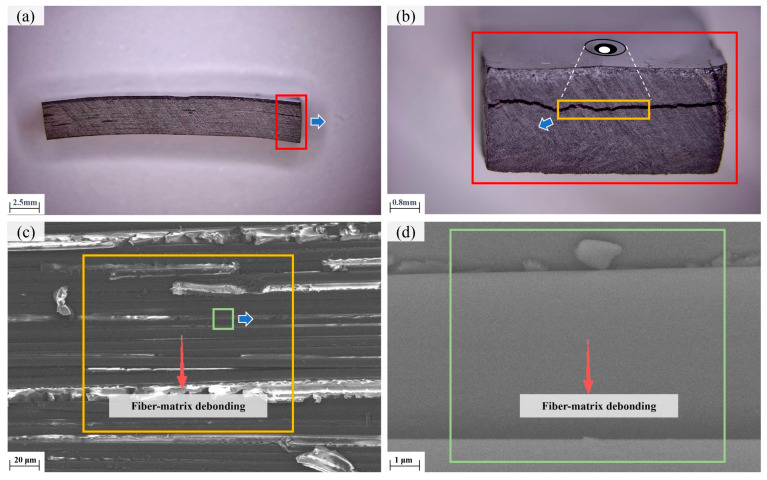
Shear failure morphology of NOL ring: (**a**) Macroscopic; (**b**) Mesoscopic; (**c**) SEM; (**d**) High magnification SEM.

Further characterization of shear fracture specimens is presented in Figure 16. Shear failure occurred predominantly along the interlaminar regions, exhibiting typical interlaminar failure characteristics. SEM observations of the delaminated interfaces reveal intact fibers with no significant fiber pull-out or fracture, indicating that shear failure is governed by fiber–resin interfacial debonding. Therefore, heating temperature affects shear performance primarily by regulating interfacial bonding quality. Within the investigated processing window, higher heating temperatures lead to improved interfacial adhesion and enhanced interlaminar shear strength.

### 4.3. Engineering Validation on Type IV Hydrogen Storage Vessels

To validate the engineering applicability and performance advantages of the dry towpreg filament winding process optimized through multi-objective optimization, this study fabricated 9 L Type IV towpreg dry filament-wound hydrogen storage vessels using the optimized process parameters (winding tension of 79 N, heating temperature of 360 °C, and winding speed of 11 m/min). For comparison, three wet filament-wound hydrogen storage vessels were also fabricated using the same winding pattern and tension scheme, with all four vessels designed to withstand a burst pressure of 60 MPa. Hydrostatic burst tests were conducted to systematically compare the burst pressure, failure modes, and mass characteristics of the vessels produced by the two different processes.

For comparison, three wet filament-wound hydrogen storage vessels were also fabricated using the same winding pattern and tension scheme, with all four vessels designed to withstand a burst pressure of 60 MPa. It should be noted that the process parameters for the wet-wound vessels were selected based on prior experience and literature [2] to ensure representative performance of the wet winding process, and have been demonstrated to achieve high fiber strength utilization in previous studies. Hydrostatic burst tests were conducted to systematically compare the burst pressure, failure modes, and mass characteristics of the vessels produced by the two different processes.

#### 4.3.1. Burst Performance and Failure Mode Analysis

**Figure 17:**
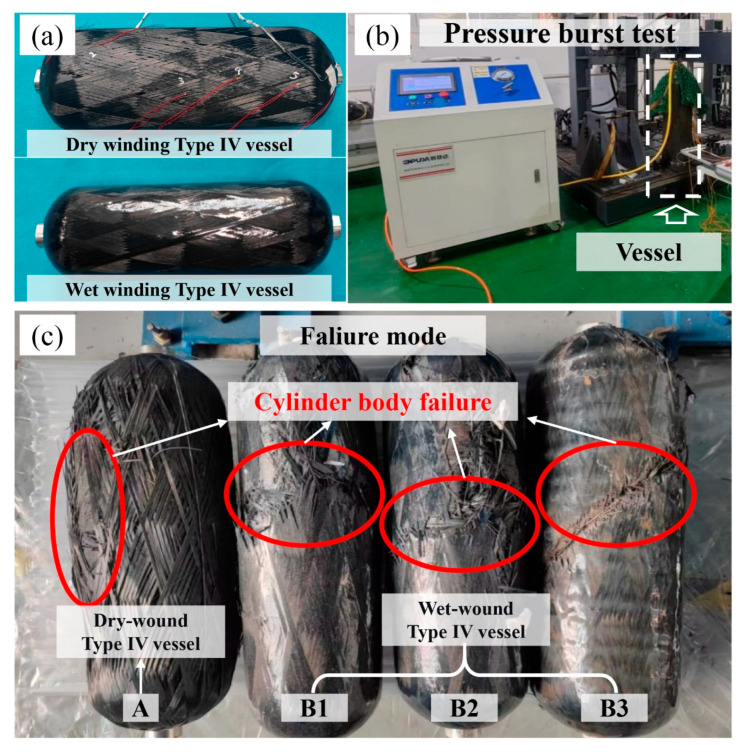
Hydrostatic burst test verification: (**a**) Wound vessel; (**b**) Hydrostatic burst test; (**c**) Failure mode.

The results of the hydrostatic burst tests are presented in Table 8. The burst pressure of the dry filament-wound vessels was 56 MPa, which is closely comparable to the average burst pressure of 56.3 MPa for the wet filament-wound vessels, indicating that the optimized dry towpreg filament winding process achieves a comparable load-bearing capacity to the conventional wet filament winding process. As shown in Figure 17c, the failure mode analysis reveals that both types of vessels exhibited failure in the cylindrical section. The burst pressure results from both processes show high consistency, with the fiber strength being effectively utilized in both cases.

#### 4.3.2. Structural Efficiency Analysis

According to the analysis in Table 8, the mass of the hydrogen storage vessels fabricated by different processes was compared. For vessels with the same winding pattern, the dry filament-wound vessels had a mass of 2915.68 g, which is approximately 15.4% lower than the average mass of the wet filament-wound vessels (3446.63 g), demonstrating a clear advantage in lightweight design.

Further evaluation of structural efficiency was conducted using the specific hydrogen storage capacity indicator (PV/M), defined as pressure×volume/mass. The PV/M value of the dry filament-wound vessels was 0.1728 MPa·L/g, significantly higher than the 0.1471 MPa·L/g of the wet filament-wound vessels. This indicates that the dry filament-wound vessels have a higher gravimetric storage ratio and improved structural efficiency.

Performance comparisons show that the burst pressure of the dry filament-wound vessels reached the same level as that of the wet filament-wound vessels. Under the same pressure rating, the dry filament-wound vessels exhibited a mass reduction of approximately 15%, along with a 17% improvement in specific hydrogen storage capacity. This advantage primarily arises from the precise control of resin content in the dry towpreg filament winding process, which avoids the local resin-rich or resin-deficient areas caused by uneven resin impregnation in the wet process, thereby increasing the fiber volume fraction [27,28]. Additionally, the optimized winding parameters not only ensure tight fiber placement but also reduce friction-induced damage. This promotes thorough resin impregnation and enhance interfacial bonding, further improving the structural efficiency of the composite materials.

Moreover, the dry towpreg filament winding process is less affected by environmental factors and offers better consistency, contributing to stable and reliable vessel performance. This study provides both theoretical insights and process guidelines for the application of dry towpreg filament winding technology in the lightweight manufacturing of high-pressure hydrogen storage vessels.

## 5. Conclusions

In this study, aiming at the efficient and high-quality fabrication of Type IV high-pressure hydrogen storage vessels, winding tension, heating temperature, and winding speed during the filament winding process were selected as the key process parameters, and a comprehensive optimization of the mechanical properties of material including tensile strength and interlaminar shear strength of NOL ring specimens was conducted. A regression model correlating the winding process parameters with the mechanical properties of NOL rings was established based on RSM. Multi-objective optimization was subsequently performed by combining the NSGA-II, and the optimal process parameter combination was determined using the TOPSIS.

Dry towpreg filament winding experiments were designed based on RSM. By combining the experimental results of tensile strength and shear strength of composite materials, a multivariate regression prediction model describing the relationship between the performance of filament-wound products and key process parameters was established. Through residual analysis and comparison between predicted values and experimental results, the regression model was verified to exhibit high accuracy and reliability.

Based on the established multivariate regression models, a sensitivity analysis was conducted to evaluate the influence of process parameters on different mechanical performance indicators of the composites. The results show that interlaminar shear strength is most significantly affected by heating temperature, followed by winding tension, and is least affected by winding speed. In contrast, tensile strength is most strongly influenced by winding tension, while the effects of heating temperature and winding speed are relatively small.

With the objectives of maximizing tensile strength and interlaminar shear strength, a multi-objective optimization of the process parameters was carried out using the NSGA-II algorithm, yielding the corresponding Pareto solution set. By applying the TOPSIS decision-making method, the optimal combination of process parameters was determined to be a winding tension of 79 N, a heating temperature of 360 °C, and a winding speed of 11 m/min. Under these conditions, the tensile strength and interlaminar shear strength of the fabricated NOL ring specimens reached 2462.2 MPa and 64.4 MPa, respectively, with prediction errors less than 0.5%, thereby validating the effectiveness of the proposed optimization method. While the model demonstrates high predictive accuracy within the studied parameter domain, its applicability outside this range warrants further investigation.

The optimized dry towpreg filament winding process was further applied to the fabrication of Type IV hydrogen storage vessels. The results indicate that the burst pressure of the dry towpreg filament wound vessel is comparable to that of vessels manufactured using the conventional wet filament winding process, while the overall mass is reduced by approximately 15%, leading to a significant improvement in gravimetric hydrogen storage capacity. The multi-objective optimization method established in this study is reliable, and can provide theoretical support and process guidance for the application of dry towpreg filament winding technology in lightweight high-pressure hydrogen storage vessels.

## Figures and Tables

**Table 1:** Range of process parameters for NOL ring.

Parameters	*A*/N	*B*/℃	*C*/(m/min)
Value range	50~110	120~360	5~17

Notes: *A* is the winding tension; *B* is the heating temperature; *C* is the winding speed.

**Table 2:** Results of the three-factor, three-level orthogonal experiment for dry towpreg filament winding process.

	*A*/N	*B*/℃	*C*/(m/min)	*R*_1_/MPa	*R*_2_/MPa
1	50	120	11	58.0	2397.5
2	50	240	5	58.8	2408.4
3	50	240	17	59.0	2403.2
4	50	360	11	62.6	2416.5
5	80	120	17	58.9	2432.4
6	80	120	5	58.7	2436.0
7	80	240	11	61.6	2445.3
8	80	240	11	61.6	2445.3
9	80	240	11	61.6	2445.3
10	80	360	17	63.9	2453.4
11	80	360	5	63.5	2463.2
12	80	240	11	61.6	2445.3
13	80	240	11	61.6	2445.3
14	110	240	17	62.4	2280.9
15	110	360	11	64.9	2288.0
16	110	240	5	62.3	2284.1
17	110	120	11	60.1	2276.2

Notes: *A* is the winding tension; *B* is the heating temperature; *C* is the winding speed; $R_{1}$ is the shear strength; $R_{2}$ is the tensile strength in the fiber direction.

**Table 3:** ANOVA for the shear strength regression model $R_{1}$.

Source	Sum of Squares	*d*_f_	Mean Square	F-Value	*p*-Value
Model	65.43	9	7.27	58.51	<0.0001
A	15.88	1	15.88	127.79	<0.0001
B	46.95	1	46.95	377.88	<0.0001
C	0.1081	1	0.1081	0.8702	0.3820
AB	0.0121	1	0.0121	0.0974	0.7641
AC	0.0000	1	0.0000	0.0002	0.9891
BC	0.0081	1	0.0081	0.0652	0.8058
$A^{2}$	0.7650	1	0.7650	6.16	0.0421
$B^{2}$	0.1791	1	0.1791	1.44	0.2689
$C^{2}$	1.50	1	1.50	12.05	0.0104
Residual	0.8697	7	0.1242		
Lack of Fit	0.8697	3	0.2899		
Pure Error	0.0000	4	0.0000		
Cor Total	66.30	16			
$R^{2}$	0.9869				
${R^{2}}_{Adj}$	0.9700				

Notes: *d*_f_ is the degree of freedom; F is ratio between interclass variance and intraclass variance; *p* is used to evaluate the significance of the model and is related to F.

**Table 4:** ANOVA for the tensile strength regression model $R_{2}$.

Source	Sum of Squares	*d*_f_	Mean Square	F-Value	*p*-Value
Model	75,159.74	9	1370.57	957.31	<0.0001
A	30,787.51	1	5052.80	7976.99	<0.0001
B	781.42	1	128.25	1519.59	<0.0001
C	59.78	1	9.81	230.20	0.0166
AB	13.25	1	2.18	32.97	0.1838
AC	0.9957	1	0.1634	138.48	0.6981
BC	9.63	1	1.58	170.72	0.2490
$A^{2}$	43,296.64	1	7105.78	43.31	<0.0001
$B^{2}$	1.95	1	0.3202	21.38	0.5892
$C^{2}$	0.3691	1	0.0606	0.1155	0.8127
Residual	42.65	7	6.09		
Lack of Fit	42.65	3	14.22		
Pure Error	0.0000	4	0.0000		
Cor Total	75,202.39	16			
$R^{2}$	0.9994				
${R^{2}}_{Adj}$	0.9987				

Notes: *d*_f_ is the degree of freedom; F is ratio between interclass variance and intraclass variance; *p* is used to evaluate the significance of the model and is related to F.

**Table 5:** Expert scoring values for different evaluation indicators.

Expert Number	Indicator 1 (Shear Strength)	Indicator 2 (Tensile Strength)
1	3	5
2	3	4
3	3	5
4	3	4
5	4	5
6	3	4
7	3	5
8	3	5
9	4	5
10	3	5

**Table 6:** Top 10 solutions in the Pareto-optimal solution set.

Ranking	Process Parameters			Evaluation Indicators		*R_m_*
	Winding Tension /N	Heat Temperature /℃	Winding Speed/(m·min^−1^)	Shear Strength /MPa	Tensile Strength /MPa	
1	79	360	11	64.21	2457.85	0.8466
2	75	360	11	64.22	2457.14	0.8458
3	83	360	12	64.16	2459.64	0.8456
4	78	360	11	64.16	2459.64	0.8456
5	81	360	11	64.31	2453.59	0.8443
6	77	360	11	64.11	2461.20	0.8435
7	71	360	10	64.27	2454.31	0.8423
8	82	360	11	64.36	2451.12	0.8406
9	76	360	12	64.07	2461.83	0.8403
10	75	360	12	64.07	2461.83	0.8403

Notes: *R_m_* is the proximity index of each non-dominated solution to the optimal level.

**Table 7:** Experimental validation results of the optimal process parameters.

Parameters	Predicted Result	Experimental Result						Error /%
		No. 1	No. 2	No. 3	No. 4	No. 5	Average Value	
Shear strength /MPa	64.2	64.8	64.2	64.7	64.0	64.1	64.4	0.31%
Tensile strength /MPa	2457.8	2468.8	2459.1	2461.8	2463.6	2458.0	2462.2	0.18%

**Table 8:** Performance comparison of hydrogen storage vessels fabricated by different processes.

No.	Winding Type	Mass/g	Pressure/MPa	PV/M(MPa·L/g)
A	Dry winding	2915.68	56	0.1728
B1	Wet winding	3410.35	53.5	0.1412
B2	Wet winding	3480.84	57.5	0.1487
B3	Wet winding	3448.70	58	0.1513

## Data Availability

The original contributions presented in this study are included in the article. Further inquiries can be directed to the corresponding authors.

## Abbreviations

The following abbreviations are used in this manuscript:

ANOVA	Analysis of variance
RSM	Response surface methodology
NSGA-II	Non-dominated sorting genetic algorithm II
TOPSIS	Technique for Order Preference by Similarity to Ideal Solution
BBD	Box–Behnken design
SEM	Scanning electron microscopy
PV/M	Pressure × volume/mass
